# Supplementary material for: Missing molybdenum and the composition of the continental crust inferred from molybdenum isotopes
Source: Nat Commun. 2025 Dec 18;16:11436. doi: 10.1038/s41467-025-66234-5 (PMC12748804; doi:10.1038/s41467-025-66234-5)
Supplement: Supplementary file 1 — Supplementary information [file 41467_2025_66234_MOESM1_ESM.pdf]

Supplementary Information for

Missing molybdenum and the composition of the continental crust inferred from molybdenum isotopes

Yamei Tian[1, 2], Feng Huang[1,3*], Jifeng Xu[1,3*], Jie Li[4], Yunchuan Zeng[1,3], Alex J. McCoy-West[2,5*]

[1] *State Key Laboratory of Geological Processes and Mineral Resources and School of Earth Science and Resources, China University of Geosciences, Beijing 100083, China*
[2] *IsoTropics Geochemistry Laboratory, Earth and Environmental Science, James Cook University, Townsville, Australia, 4811*
[3] *Frontiers Science Center for Deep-time Digital Earth, China University of Geosciences, Beijing 100083, China*
[4] *State Key Laboratory of Deep Earth Processes and Resources, Guangzhou Institute of Geochemistry, Chinese Academy of Sciences, Guangzhou 510640, China*
[5] *Economic Geology Research Centre, James Cook University, Townsville, Australia, 4811*

* fenghuang@cugb.edu.cn; jifengxu@cugb.edu.cn; alex.mccoywest@jcu.edu.au

Content of this file:
Supplementary Text
Supplementary Figures 1-11
Supplementary Tables 1-7
Supplementary References

Supplementary Text

Geological setting and samples of Gangdese arc crustal section

The Gangdese arc lies within the Lhasa Terrane in southern Tibet (Supplementary Fig. 1). Gangdese arc magmatism started in the Triassic and persisted until the Paleocene with three magmatic flare-up events at 200–160 Ma, 120–105 Ma and 100–80 Ma[1-3], which are likely related to the variations of subduction stage of the Neo-Tethyan oceanic slab[4,5]. The Gangdese arc crustal section is exposed in the southeastern Lhasa Terrane, which is a continuous exposure of a continental arc crust (Supplementary Fig. 1c) ranging in depth from 42 km to 5 km[6]. In the southeastern region of the Lhasa Terrane (Supplementary Fig. 1c), the Lilong lower crustal section is exposed and is structurally bounded by the Dongjiu–Milin shear zone[7,8]. Here, the lower crust of the Gangdese arc in the hanging wall is juxtaposed against the Himalayan sequences in the footwall (Supplementary Fig. 1c).

The eastern Gangdese arc comprises the Nyingchi Complex, the Kanniang Complex, the Lilong Complex, and the Wolong batholith, arranged from northeast to southwest (Supplementary Fig. 1c). The Nyingchi Complex underwent granulite- or amphibolite-facies metamorphism during the Cenozoic (ca. 55–25 Ma)[9,10]. The Kanniang Complex, representing the deepest exposed level of the Gangdese arc crust, consists mainly of garnet-bearing gabbros[11,12]. The Lilong Complex includes a range of arc-related ultramafic to intermediate intrusions, with high-temperature mafic-intermediate rocks that have led researchers[2,13] to suggest metamorphism associated with ridge subduction and oceanic slab rollback. The Wolong batholith, primarily composed of Late Cretaceous (84–74 Ma) granodiorite and granite, is characterized by low Mg#, along with high Sr/Y and La/Yb ratios, indicative of formation by partial melting of thickened lower crust within the Gangdese arc[14,15]. The Gangdese arc deeper crustal section displays depleted Sr–Nd isotope compositions[6,9], which reflect a significant influx of juvenile material from the depleted mantle.

The Gangdese arc crust samples in this study are granites, quartz diorites, tonalites, gabbros, hornblendite and pyroxene hornblendites, which are collected at Wolong, Lilong and Milin area in the eastern part of Gangdese arc crustal section (Supplementary Fig. 1c). The granites (Supplementary Fig. 2a) are composed of plagioclase (∼30–40 vol.%), K-feldspar (∼25–30 vol.%), quartz (∼20–30 vol.%) and minor biotite (∼3–5 vol.%). The quartz diorites are composed of plagioclase (45–50 vol.%), amphibole (20–35 vol.%), quartz (5–10 vol.%), biotite (4–8 vol.%), K-feldspar (1–6 vol.%), and clinopyroxene (2–6 vol.%). Based on mineral grain size, two textural varieties can be distinguished (Supplementary Figs. 2b-c): (1) a medium-coarse grained type with dominant grain sizes > 0.2 mm (maximum dimensions reaching 0.5 mm in width and 2 mm in length), and (2) a fine-grained variety characterized by plagioclase laths measuring 0.05–0.2 mm in length and other minerals typically < 0.2 mm. The tonalites (Supplementary Fig. 2d) are made up of plagioclase (50–60 vol.%), amphibole (10–20 vol.%), K-feldspar (2–5 vol.%), quartz (10–20 vol.%), and biotite (2–6 vol.%). The gabbros (Supplementary Fig. 2e) display a heteradcumulate texture where poikilitic amphibole (5–15 vol.%) encloses plagioclase (35–45 vol.%) and clinopyroxene (40–50 vol.%). The hornblendites and pyroxene hornblendites (Supplementary Figs. 2f-g) are mainly composed of hornblende (50–90 vol.%) with subordinate clinopyroxene (5–25 vol.%), while the latter contains higher volume of clinopyroxene (15–25 vol.%). Both pyroxene and hornblende grains exhibit coarse-grained textures with minimum grain sizes exceeding 1 mm. The detailed petrographic characteristics of these rocks have been well documented in previous studies[2,6].

All samples exhibit fresh mineral surfaces without visible alteration features. They display consistent rare earth element (REE) patterns (Supplementary Fig. 3), and mobile elements (e.g., Rb, Ba) show no significant depletion. These features collectively demonstrate that the chemical compositions of these samples remain pristine, unaffected by post-magmatic low-grade metamorphism or weathering processes (Supplementary Fig. 4).

These felsic, intermediate, and ultramafic-mafic rocks display distinct geochemical signatures (Supplementary Figs. 3 and 5). Granites (SiO_2 = 67.8–68.8 wt.%) are characterized by strongly fractionated chondrite-normalized REE patterns with light rare earth element (LREE) enrichment (Supplementary Fig. 3b). Quartz diorites and tonalites (SiO_2 = 53.1–57.3 wt.%) exhibit prominent LREE enrichment, moderate HREE depletion, and relatively flat MREE segments (Supplementary Fig. 3d). In contrast, hornblendites and pyroxene hornblendites (SiO_2 = 41.6–44.8 wt.%) exhibit convex-upward chondrite-normalized REE patterns (Supplementary Fig. 3f). The Gangdese arc crust samples exhibit enrichment in large ion lithophile elements (LILEs) and depletion in high field strength elements on primitive mantle-normalized diagrams (Supplementary Fig. 3).

Assessment of alteration within the Gangdese arc crustal samples

It is important to ensure that the samples are unaltered and not affected by secondary processes. During sampling all reasonable precautions were taken to obtain the most pristine samples possible. Low-temperature process of igneous rocks can cause significant Mo isotope fractionation, with light Mo preferentially adsorbed by weathering products (e.g., Fe-Mn oxides, clay minerals) or organic matter, while heavy Mo is more readily transported into water[16]. During the weathering of rocks, and depending on factors such as pH and redox conditions, primary minerals are progressively altered and dissolved, and secondary minerals are formed[17,18]. The granites are mainly composed of quartz, plagioclase, biotite and hornblende. The medium-coarse-grained quartz diorites, fine-grained quartz diorites and tonalites are mainly composed of plagioclase, hornblende, biotite, and quartz. The main minerals in the hornblendites and pyroxene hornblendites are hornblende and pyroxene, while the gabbros are mainly composed of pyroxene, plagioclase and amphibole. All these minerals are pristine magmatic phases, and display no significant signs of alteration. In addition, investigation of the degree of chemical alteration within the Gangdese arc crustal samples based on their geochemistry compositions has been undertaken (Supplementary Fig. 4). All of samples shows relatively low ($\leq$ 1.9 wt.%) LOI (loss on ignition) values, a potential indicator of secondary hydrous mineral formation (Supplementary Fig. 4a). An alternative approach to quantifying alteration, less sensitive to initial bulk rock composition, has been developed by Ohta and Arai[19]. Their mafic-felsic-weathered (MFW) diagram (Supplementary Fig. 4b) uses principal component analyses of 8 major elements (SiO_2, TiO_2, Al_2O_3, $Fe_2O_3^T$, MgO, CaO, Na_2O and K_2O) to assess the consequences of alteration. All of the Gangdese arc crustal samples show little evidence for chemical weathering given the plot near the unaltered igneous rock array.

Evaluating the Mo isotope composition of the UCC

Given that the UCC is volumetrically dominated by felsic intrusive rocks, with sedimentary components accounting for less than ca. 8%[20-23], this study employs the $\delta^{98/95}$Mo of intrusive rocks to represent that of the upper continental crust. However, the Mo isotopic signatures of surficial sediments[20] and weathering products[16,24] must also be considered. Averages of insoluble element

(e.g., Mo) compositions in glacial deposits, like diamectites which comprise a mixture of igneous and weathered crustal materials, can be used to infer upper crustal composition[20,24]. Loess and clay represent typical aeolian sediments[16]. During weathering, Mo is initially liberated from primary minerals and subsequently undergoes partial adsorption and desorption onto weathering products (e.g., Fe–Mn oxides, loess and clay minerals), accompanied by Mo isotope fractionation[16]. Despite these surficial deposits only accounting for a small volume of the UCC, they exert a dominant control on trace element enrichment and depletion, particularly in loess and clay[20]. To better constrain the potential contribution from the sedimentary archives to the $\delta^{98/95}$Mo of the UCC, we compiled Mo concentration and $\delta^{98/95}$Mo from glacial diamictites, loess, and clay[16,24]. Mo concentrations exhibit a wide range without a clear central peak (0.19–2.97 µg/g), with a median value of 0.72 µg/g (Supplementary Table 3). The median $\delta^{98/95}$Mo value is −0.070 ± 0.119‰ (95% s.e.; n = 45; ± 0.790‰, 2 s.d.) (Supplementary Fig. 8 and Supplementary Table 4). Notably, the median $\delta^{98/95}$Mo of these sedimentary archives closely resemble those of the compiled felsic intrusive rocks, suggesting that the $\delta^{98/95}$Mo of intrusive rocks can reliably be used to represent that of the UCC.

Supplementary Figures and Tables

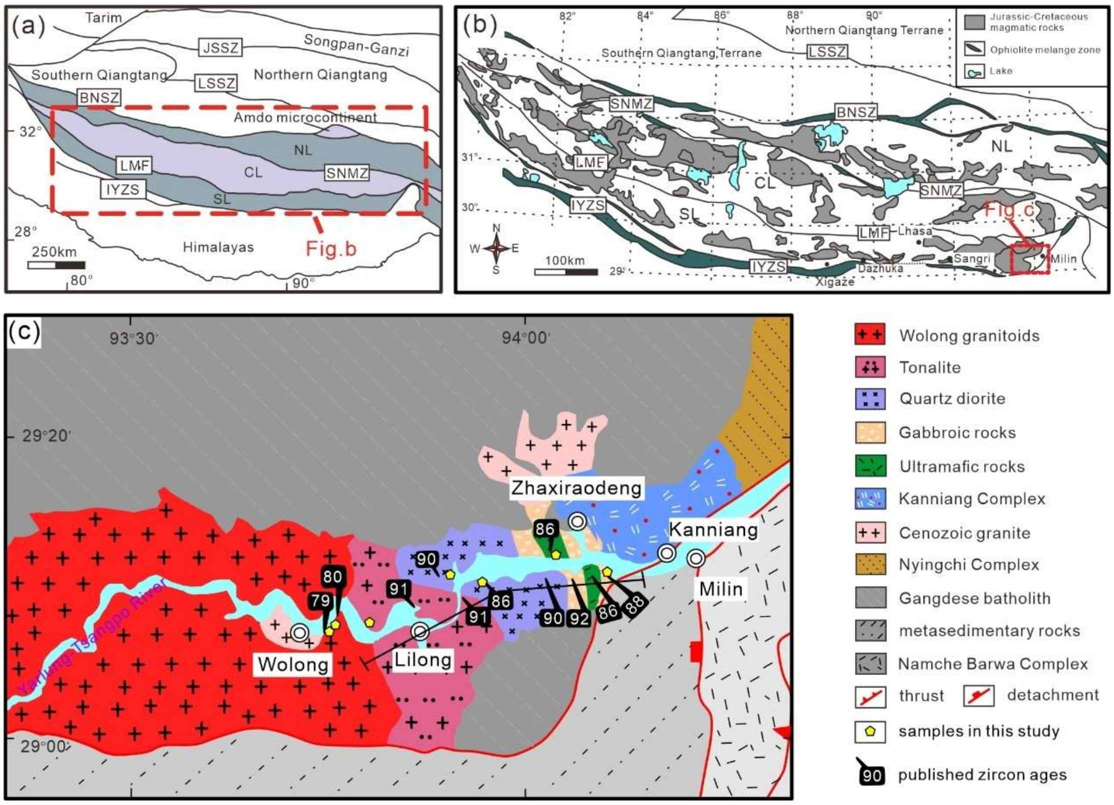

Supplementary Figure 1: Sketch maps of the Tibetan Plateau, Lhasa Terrane, and study area. (a) The Lhasa Terrane in the context of the Tibetan Plateau. (b) Geological map of the Lhasa Terrane, modified from Tian et al.[25]. (c) Geological map of the Gangdese arc crust section showing the sample locations, modified from Guo et al.[6]. Published zircon U–Pb ages are from Guo et al.[6] and references therein. Abbreviations: JSSZ—Jinsha suture zone; LSSZ—Longmu Tso–Shuanghu suture zone; BNSZ—Bangong-Nujiang suture zone; NL—northern Lhasa Terrane; CL—central Lhasa Terrane; SL—southern Lhasa Terrane; SNMZ—Shiquan River–Nam Tso mélange zone; LMF—Luobadui-Milashan fault; IYZS—Indus–Yarlung–Zangbo suture zone.

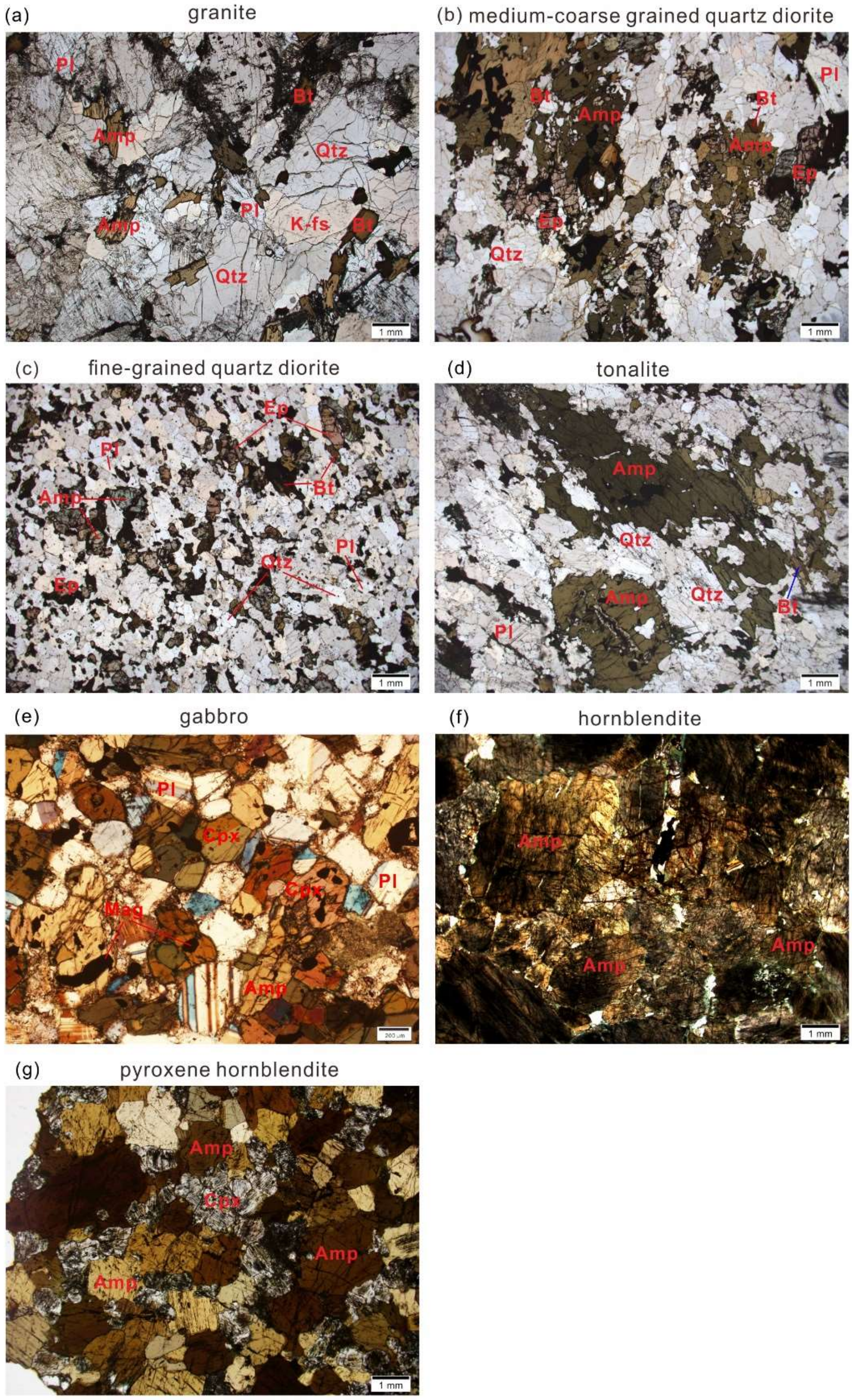

Supplementary Figure 2: Photomicrographs of the typical lithologies from various parts of the Gangdese arc crust section. (a) Granite belongs to felsic rocks, representing the upper continental crust (UCC); (b-d) Quartz diorite and tonalite belong to the intermediate intrusive rocks, representing the middle continental crust (MCC); (e-g) Gabbro, hornblendite and pyroxene hornblendite belong to ultramafic-mafic intrusive rocks, representing the lower continental crust (LCC). Mineral abbreviations: Amp = amphibole, Bt = biotite, Cpx = clinopyroxene, Ep = epidote, Qtz = quartz, Pl = plagioclase, Mag = Magnetite.

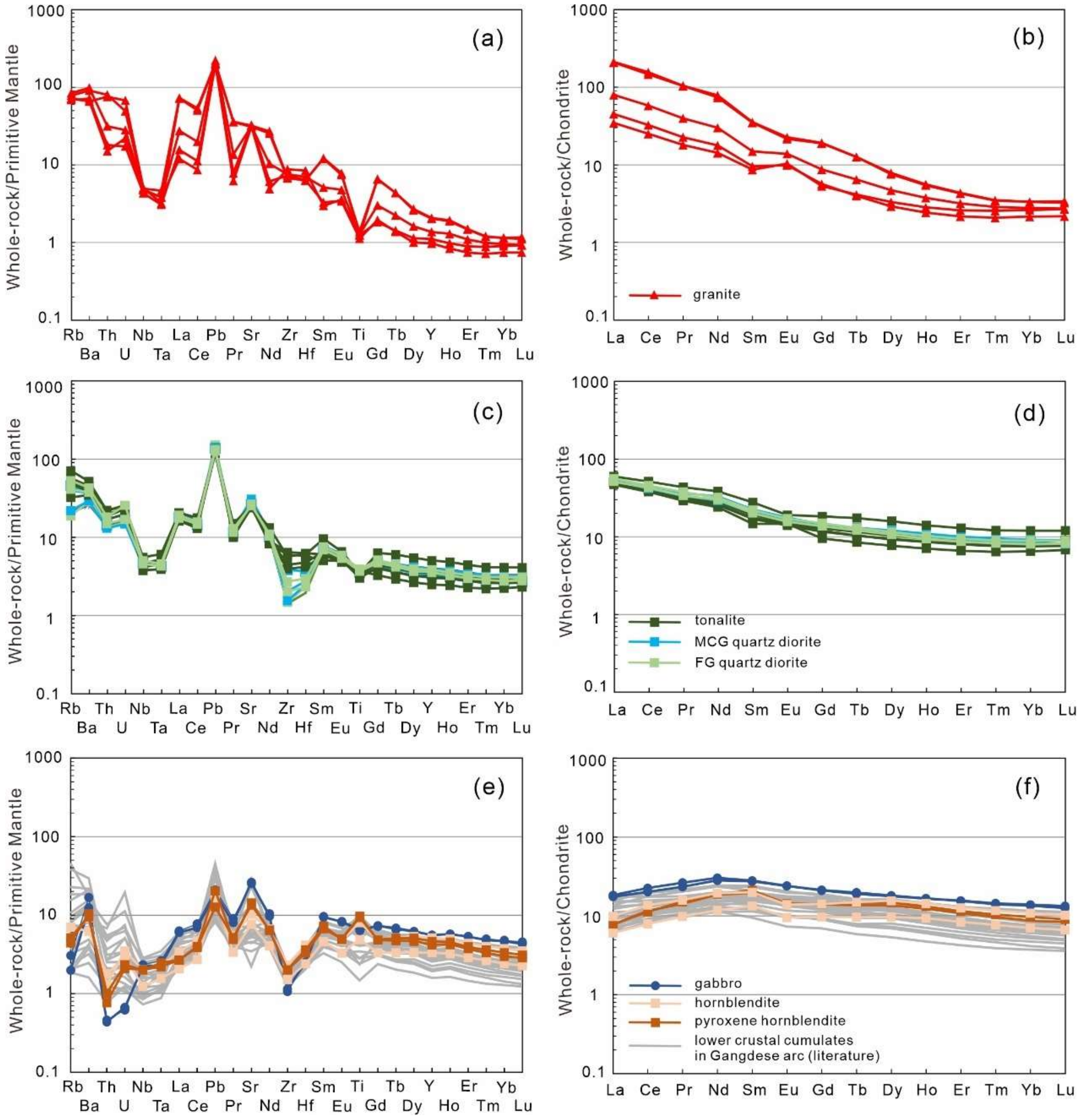

Supplementary Figure 3: Chondrite-normalized REE and primitive-mantle-normalized trace element patterns for the felsic (a–b), intermediate (c–d), and ultramafic–mafic (e–f) samples from the Gangdese arc crust. Chondrite and primitive mantle normalization values are from Sun and McDonough[26]. The lower crustal cumulate data from the Gangdese arc are sourced from Sun et al.[27]. Felsic, intermediate, and ultramafic–mafic samples from the Gangdese arc crust display distinct trace element signatures. Abbreviations: MCG = medium-coarse grained; FG = fine-grained.

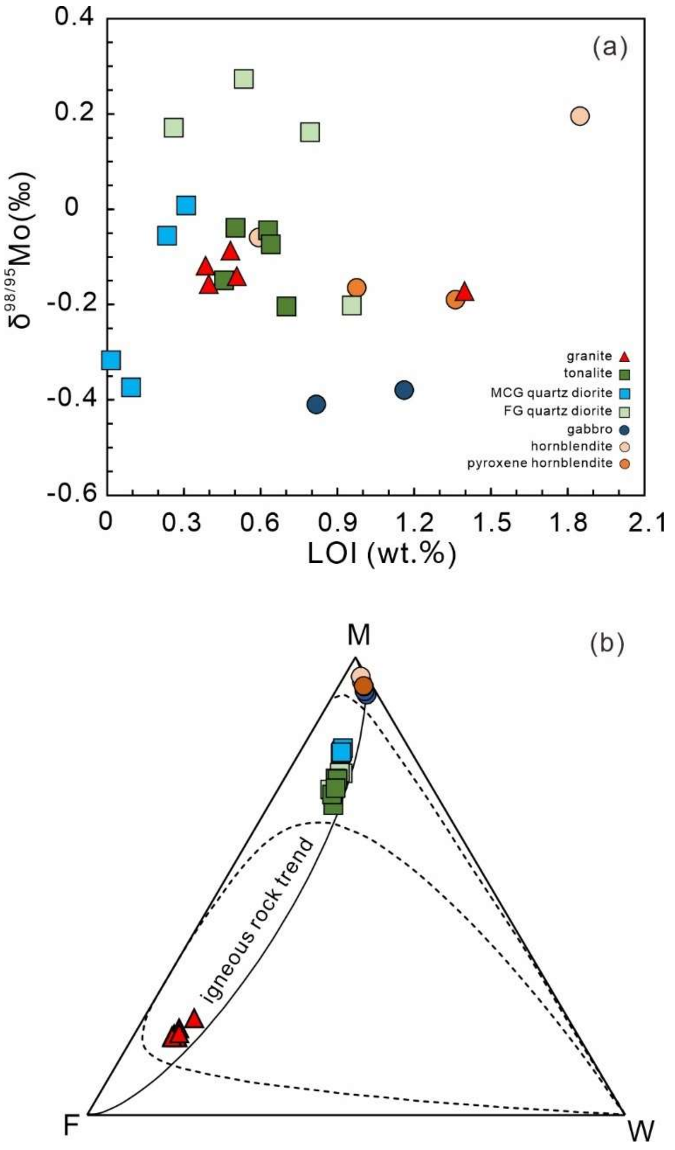

Supplementary Figure 4: An assessment of the extent of alteration within the Gangdese arc crust section samples. (a) Plot of LOI (wt.%) versus $\delta^{98/95}$Mo (‰). (b) MFW ternary plot after Ohta and Arai[19]. All samples from the Gangdese arc crustal section align closely with the igneous rock trend, indicating they are unaltered. Abbreviations: MCG = medium-coarse grained; FG = fine-grained.

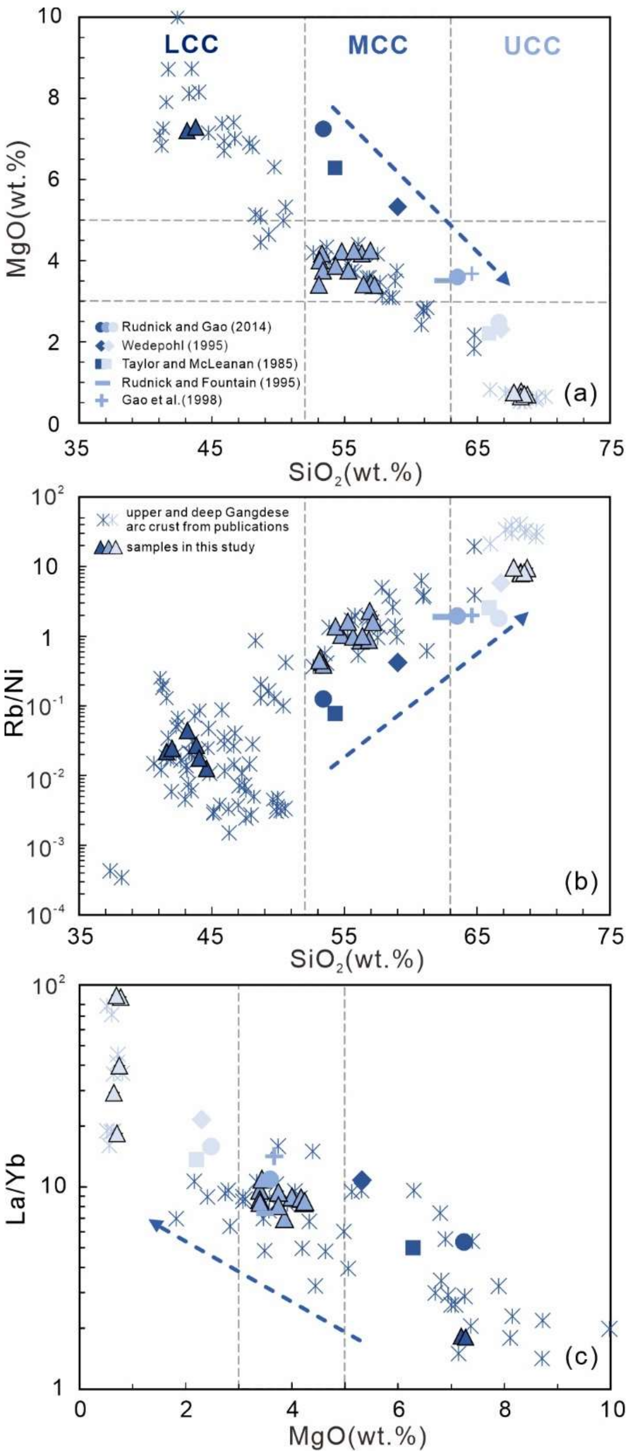

Supplementary Figure 5: Diagram of SiO_2 versus MgO (a), SiO_2 versus Rb/Ni (b) and MgO versus La/Yb (c) for deep (lower and middle) to upper in Gangdese arc crustal section and various estimates of the continental crust. Shading ranging from dark blue, blue, to light blue, represent the estimated concentrations of the lower continental crust (LCC) middle continental crust (MCC) and upper continental crust (UCC), respectively. Circles, diamonds and squares represent the estimated elemental concentrations for each crustal layer from Rudnick and Gao[20], Wedepohl[28] and Taylor and McLennan[29], respectively; short lines and plus signs represent the estimates of MCC from Rudnick and Fountain[30] and Gao et al.[31], respectively. Data of the deep and upper Gangdese arc crust are from Guo et al.[6] and Sun et al.[27].

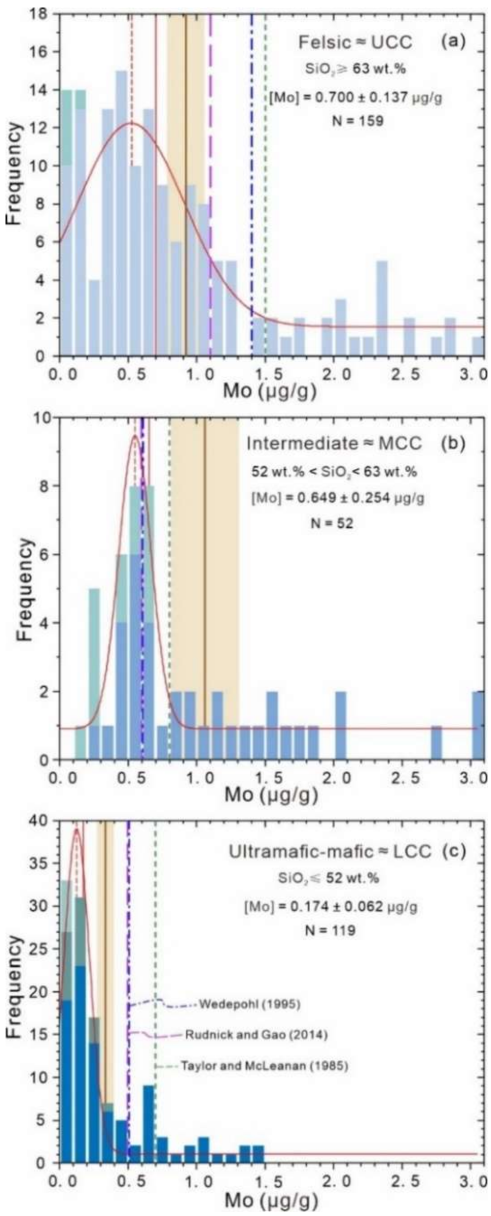

Supplementary Figure 6: Histograms of Mo concentrations comparing the compositional distribution of intrusions with $SiO_2 \geq 63$ wt.% (a), 52 wt.% < SiO_2 < 63 wt.% (b), and $SiO_2 \leq$ 52 wt.% (c). Data are compilation (Supplementary Table 3) of new samples measured herein (light green), published Gangdese arc section data (c: dark green) and other previously published data (blue). Purple, brilliant blue and green dotted lines represent the estimated Mo concentrations by Rudnick and Gao[20], Wedepohl[28] and Taylor and McLeanan[29], respectively (Supplementary Table 5). The brown lines represent the arithmetic average Mo concentrations of the different groups and the light brown bars represent 95% standard error. Red curve overlying the histogram represent relative probability density plots. Red solid lines and red dashed lines represent median and relative probability values, respectively. Mo concentration and uncertainty in each panel represent median and 95% standard error. Arithmetic average Mo concentration for each group far exceeds the median and highest probability value due to the presence of anomalously high Mo concentrations which skew the mean. The median and highest probability value of Mo concentration are significantly lower than those estimated previously[20,29]. Felsic, intermediate, and ultramafic–mafic samples represent the upper, middle and lower continental crust (i.e. UCC, MCC and LCC), respectively.

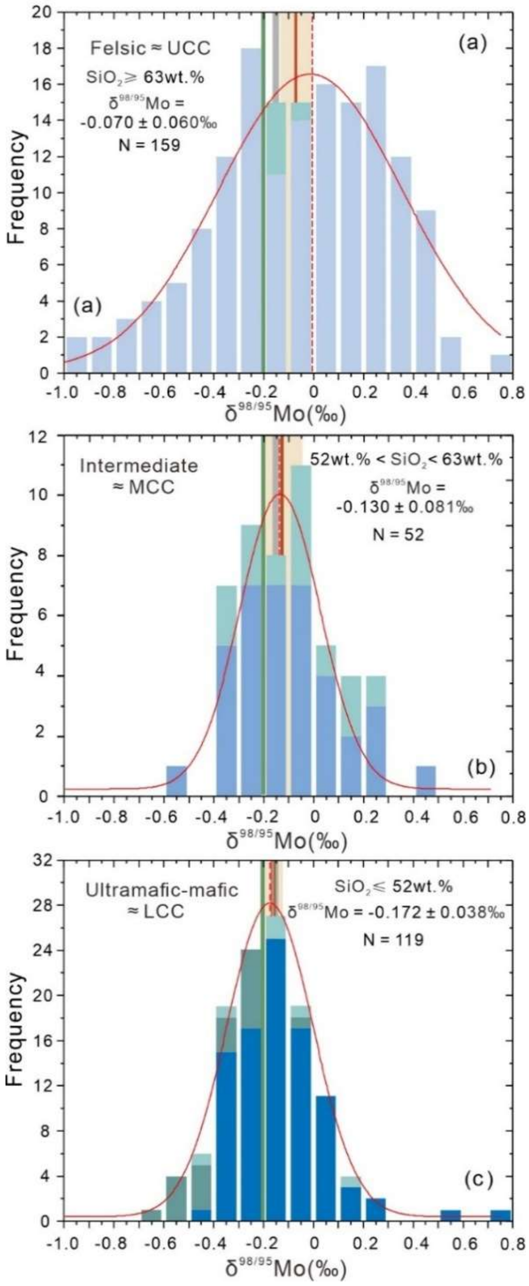

Supplementary Figure 7: Histograms comparing the Mo isotope composition ($\delta^{98/95}$Mo) of intrusive rocks with $SiO_2 \geq 63$ wt.% (a), 52 wt.% < SiO_2 < 63 wt.% (b), and $SiO_2 \leq 52$ wt.% (c). Felsic, intermediate, and ultramafic–mafic samples represent the upper, middle and lower continental crust (i.e. UCC, MCC and LCC), respectively. Data are compilation (Supplementary Table 4) of new samples measured herein (light green), published Gangdese arc section data (c: dark green) and other published data (blue). Green and gray bars represent the $\delta^{98/95}$Mo of the depleted mantle (−0.204 ± 0.008‰[32]) and bulk silicate earth (−0.154 ± 0.013‰[32]), respectively. The brown lines represent the arithmetic average $\delta^{98/95}$Mo of intrusions within each compositional group, with the light brown bars representing 95% standard error. $\delta^{98/95}$Mo and uncertainty in every panel represent the median and the 95% standard error. Red curve overlying the histogram represent relative probability density plots. Red solid lines and red dashed lines represent median and relative probability values, respectively. For isotope composition median and mean values are extremely similar, unlike Mo concentration data (see Supplementary Fig. 6).

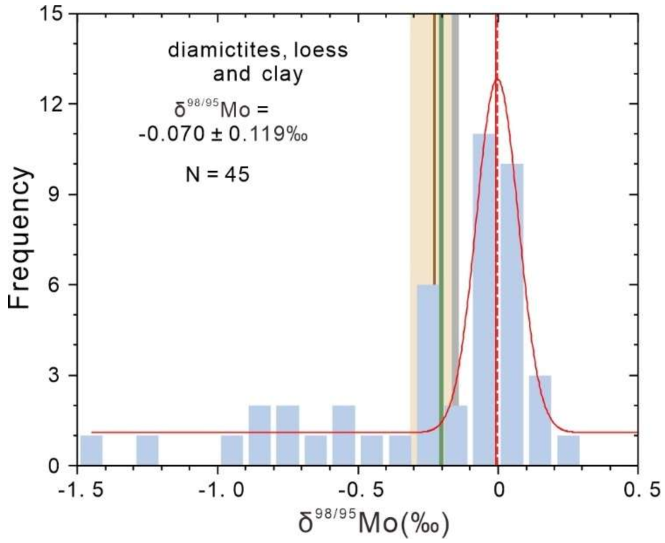

Supplementary Figure 8: Histograms of $\delta^{98/95}$Mo comparing the diamictites, clay and loess. The brown lines represent the arithmetic average $\delta^{98/95}$Mo ($\delta^{98/95}$Mo = −0.226‰), with the light brown bars represent 95% standard error. Green and gray bars represent the $\delta^{98/95}$Mo of the depleted mantle (−0.204 ± 0.008‰[32]) and bulk silicate earth (−0.154 ± 0.013‰[32]). $\delta^{98/95}$Mo and uncertainty in the panel represent the median and 95% standard error. Red curve overlying the histogram represent relative probability density plots. Red solid lines and red dashed lines represent median and relative probability values, respectively.

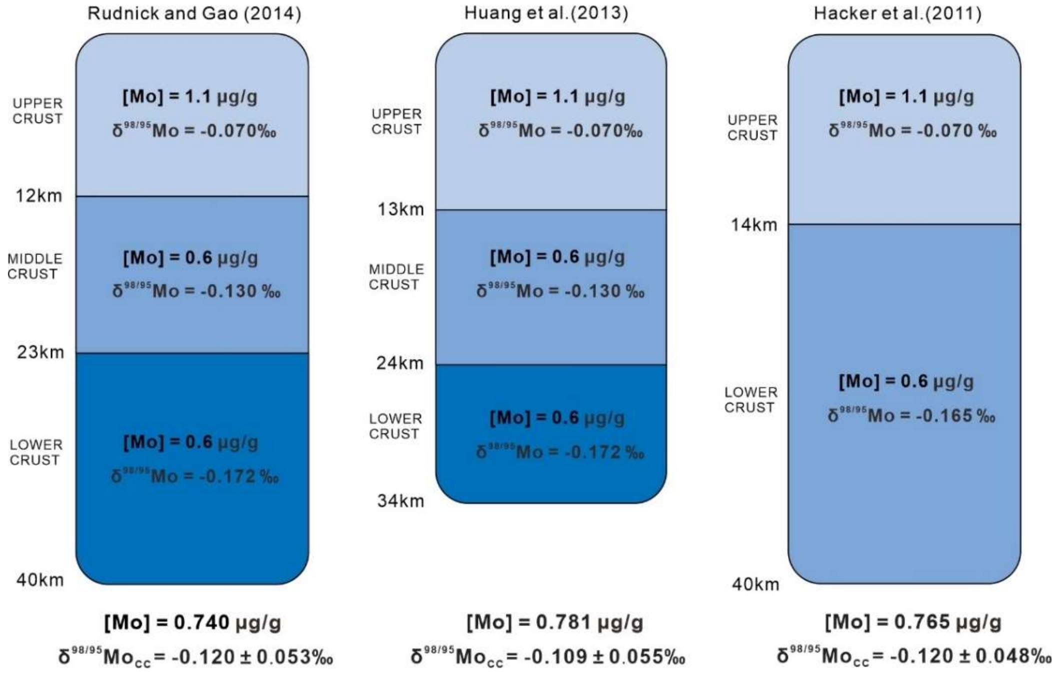

Supplementary Figure 9: Calculated Mo concentration and isotope compositions of the bulk continental crust (CC) based on different crustal depth models. Shown here Mo concentrations derived from Rudnick and Gao[20] and median $\delta^{98/95}$Mo based on the compilation herein. The thicknesses of the layers are scaled proportionally[20,33,34]. Uncertainties on the $\delta^{98/95}$Mo of the continental crust are 95% standard error. When using the same Mo concentration, different crustal depth models yield similar $\delta^{98/95}$Mo values for the bulk continental crust, alternative models are shown in Supplementary Fig. 10.

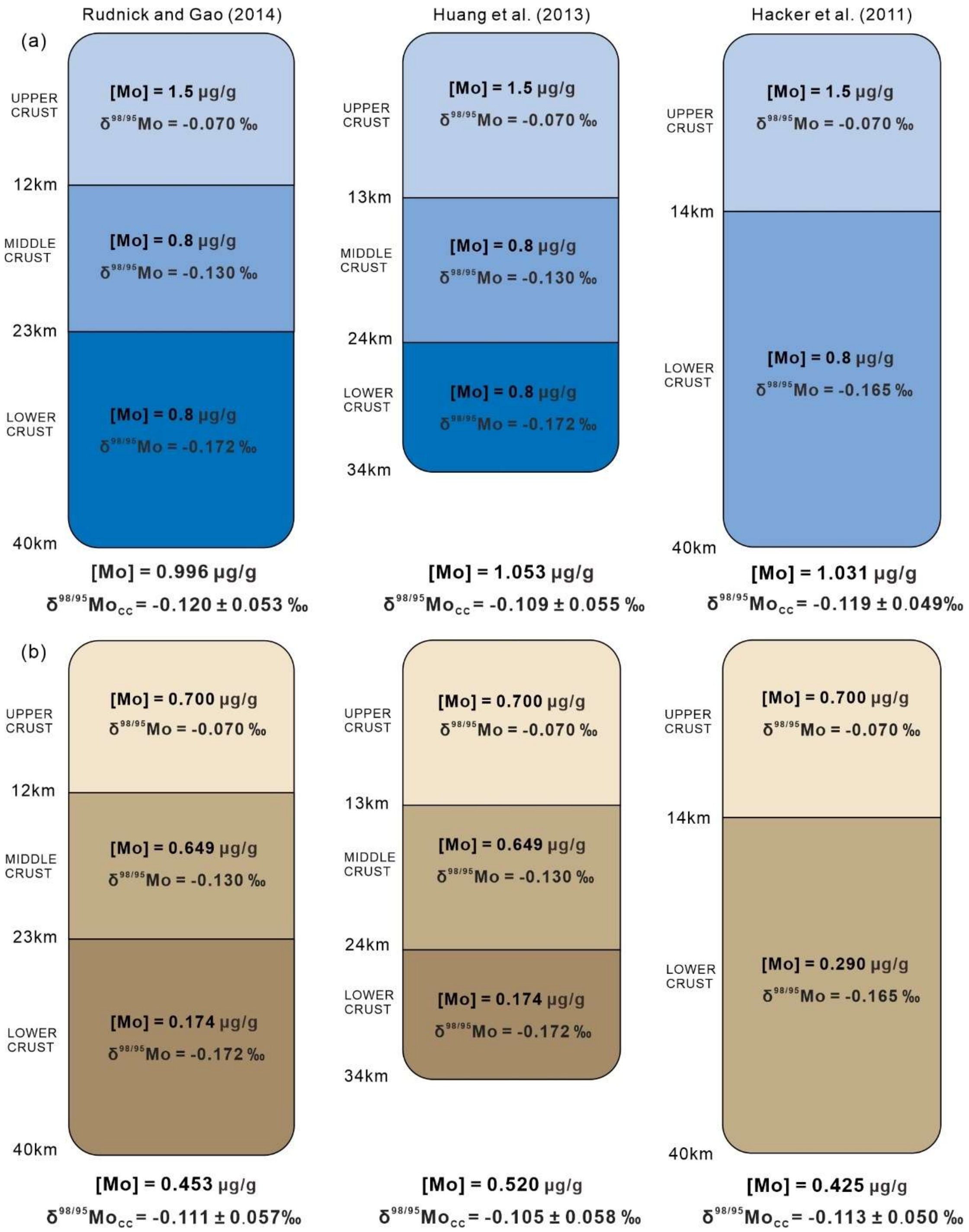

Supplementary Figure 10: Calculated composition of the bulk continental crust using different crustal depth models and alternative concentration estimates. Mo concentrations come from Taylor and McLennan[29] in (a) and the median [Mo] of intrusions compiled herein in (b). $\delta^{98/95}$Mo values come from the median of the compilation herein. Uncertainties on the $\delta^{98/95}$Mo for continental crust are 95% standard error. The thicknesses of the crustal layers are proportionally scaled[20,33,34]. The lower concentrations in the intrusion-based model result in a marginally heavier $\delta^{98/95}$Mo for the bulk continental crust.

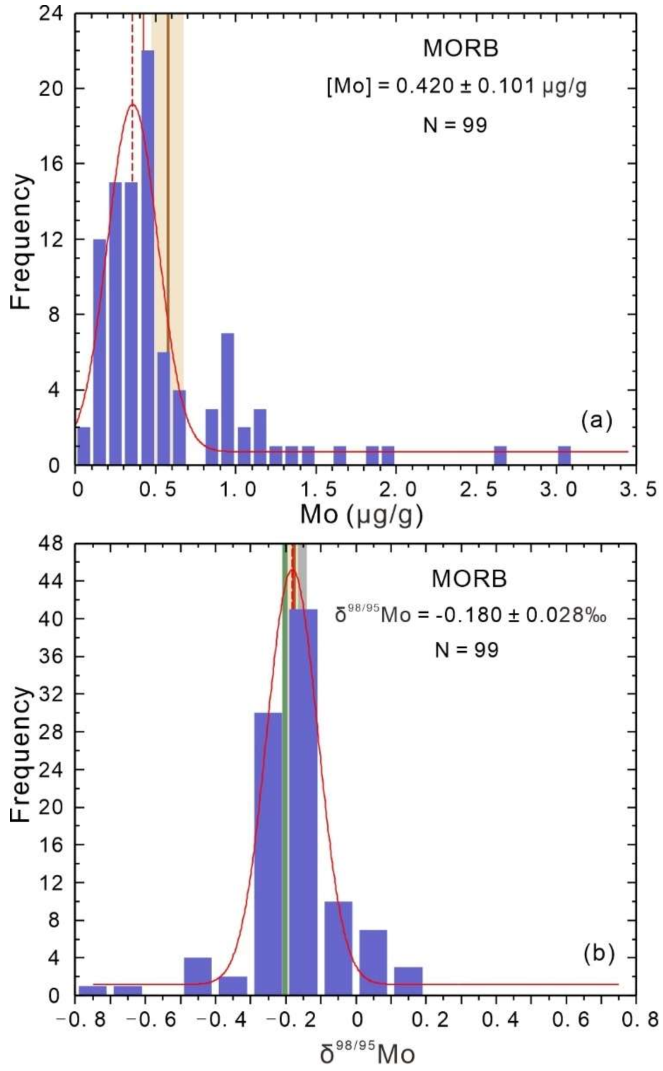

Supplementary Figure 11: Histograms of Mo concentration (a) and $\delta^{98/95}$Mo (b) comparing the compositional distribution of mid-ocean ridge basalts (MORB). The brown lines represent the arithmetic average Mo concentration and $\delta^{98/95}$Mo of MORB, with the light brown bars represent 95% standard error. Green and gray bars represent the $\delta^{98/95}$Mo of the depleted mantle (−0.204 ± 0.008‰[32]) and bulk silicate earth (−0.154 ± 0.013‰[32]). Mo concentration, or $\delta^{98/95}$Mo and uncertainty in every panel represent the highest probability value and 95% standard error. Red curve overlying the histogram represent relative probability density plots. Red solid lines and red dashed lines represent median and relative probability values, respectively.

Supplementary Table 1: Major and trace elemental and isotopic data of Gangdese arc crust section samples.

Sample No.	20WL-01	20WL-02	20WL-04	20WL-07	20WL-09	20LL-01	20LL-03	20LL-05	20LL-07	20LL-09	20LL-10	20LL-13	20LL-16
Rock type	granite					medium-coarse grained quartz diorite				fine-grained quartz diorite			
Location	Wolong					Lilong				Lilong			
Crustal layer	UCC					MCC							
Major elements (wt. %)													
SiO_2	68.31	68.79	68.30	68.56	67.78	54.79	56.30	55.71	56.95	53.30	53.14	53.37	53.07
TiO_2	0.30	0.27	0.24	0.27	0.28	0.85	0.80	0.77	0.71	0.78	0.75	0.77	0.75
Al_2O_3	16.20	16.55	16.76	16.37	16.58	17.36	17.84	18.05	18.51	19.19	19.39	19.22	19.30
$Fe_2O_3^T$	2.80	2.51	2.36	2.40	2.60	8.99	8.39	8.00	7.34	8.55	8.45	8.71	8.47
MnO	0.07	0.06	0.06	0.06	0.06	0.14	0.14	0.13	0.11	0.15	0.15	0.15	0.15
MgO	0.78	0.70	0.66	0.72	0.76	4.17	4.00	3.75	3.40	4.22	4.17	4.23	4.24
CaO	3.39	3.43	3.53	3.50	3.39	7.23	7.43	7.35	7.28	8.37	8.36	8.49	8.41
Na_2O	4.35	4.69	4.93	4.84	4.70	3.62	3.82	3.83	4.02	4.24	4.27	4.25	4.23
K_2O	2.14	2.39	1.99	1.98	2.49	1.33	1.15	1.17	1.14	0.82	0.83	0.78	0.82
P_2O_5	0.15	0.13	0.13	0.14	0.15	0.24	0.24	0.22	0.22	0.24	0.22	0.23	0.23
LOI	1.40	0.38	0.48	0.51	0.40	0.79	0.26	0.96	0.53	0.23	0.09	0.02	0.31
Total	100.03	100.08	99.59	99.50	99.37	99.66	100.36	100.08	100.34	100.22	99.95	100.22	100.11
Mg#	35.6	35.6	35.5	37.1	36.5	47.9	48.6	48.1	47.8	49.5	49.4	49.0	49.8
Trace elements (μg/g)													
Li	11.7	14.6	12	12.7	13.8	13.7	8.56	17.6	8.55	9.43	9.71	9.93	9.56
Be	1.44	1.28	1.28	1.19	1.27	0.92	0.79	0.91	0.97	1.00	0.92	0.94	1.00
Sc	9.34	9.49	9.13	8.93	9.4	23.6	22.2	21.3	19.4	24.7	23.9	24.2	24
V	32.5	28.8	27.2	26.5	29.5	175	165	172	169	185	168	189	167
Cr	2.08	1.71	1.47	1.57	1.6	36.8	27	24.8	24	22.6	21.6	22.7	22.1

Co	4.74	4.25	4.14	4.24	4.48	26.6	24.6	22.8	21.9	25.7	24.8	26.7	26.3
Ni	5.58	5.28	5.43	5.44	5.52	32.9	30.8	30.6	28.7	32.4	30.9	32.9	32.9
Cu	9.01	9.22	7.79	9.4	11.9	60.5	74.9	64.9	59.2	31.2	23.1	25.4	24.8
Zn	53.5	47.5	45.3	47.9	50.9	99.1	92.1	84.6	80	93.8	91.5	96.9	92.8
Ga	18.8	18.0	17.5	16.9	17.2	19.1	18.6	18.4	19.1	19.9	19.3	20.3	20.0
Ge	0.93	0.90	0.77	0.76	0.79	1.21	1.14	1.10	1.03	1.20	1.13	1.22	1.19
As	1.03	0.46	0.25	0.95	0.32	1.78	1.97	1.40	1.73	1.32	1.23	2.23	1.48
Rb	46	49.6	43.6	44.4	53.2	33.6	26	29.2	24.7	13	14.3	12	13.9
Sr	650	674	690	660	662	534	559	556	600	649	630	656	640
Y	9.41	9.26	4.42	5.04	6.22	17.5	16.2	15.2	14.8	18.1	16.4	17.6	17.8
Zr	75.2	77.3	81.4	98.3	84.6	30.3	22.7	43	22.8	17.4	15.9	16.5	17.4
Nb	3.59	3.51	3.08	3.38	3.39	3.58	3.26	3.36	3.06	3.29	3.02	3.18	3.04
Mo	0.179	0.096	0.108	0.087	0.146	0.346	0.594	0.527	0.479	0.735	0.730	0.727	0.701
Ag	0.138	0.141	0.126	0.144	0.147	0.168	0.158	0.158	0.151	0.148	0.128	0.145	0.132
Cd	0.031	0.032	0.027	0.028	0.029	0.094	0.090	0.092	0.087	0.111	0.107	0.109	0.101
In	0.022	0.019	0.015	0.015	0.018	0.051	0.047	0.046	0.041	0.052	0.050	0.054	0.050
Sn	0.692	0.680	0.568	0.636	0.699	0.960	0.815	0.879	0.751	0.913	0.769	0.832	0.806
Sb	0.074	0.087	0.042	0.032	0.025	0.101	0.096	0.104	0.078	0.080	0.085	0.084	0.427
Cs	0.95	1.12	1.08	1.26	1.30	1.79	1.29	1.49	1.31	0.67	0.71	0.66	0.86
Ba	459	633	499	467	685	300	261	249	260	183	188	199	204
La	49.1	50	10.7	8.19	18.8	13.2	12.1	12.5	12.8	13.5	12.5	13.2	13.1
Ce	89.3	95	19.9	15.3	35.3	27.8	26.1	25.7	26.8	29	25.4	27	27.7
Pr	9.82	9.9	2.15	1.71	3.77	3.56	3.2	3.2	3.23	3.51	3.22	3.45	3.47
Nd	34.4	36.6	8.28	6.63	14	14.9	14	13.7	13.9	15.5	13.6	14.3	15.4
Sm	5.33	5.41	1.46	1.32	2.27	3.36	3.07	3.02	3.09	3.44	3.1	3.39	3.43
Ti	1781	1603	1460	1612	1651	5089	4790	4626	4264	4672	4497	4639	4513

Eu	1.25	1.31	0.574	0.6	0.802	0.99	0.905	0.935	0.962	0.989	0.976	1	1.02
Gd	3.88	3.93	1.16	1.09	1.79	3.06	2.8	2.77	2.56	2.95	2.83	2.92	2.98
Tb	0.47	0.471	0.149	0.154	0.241	0.483	0.452	0.428	0.418	0.493	0.457	0.491	0.487
Dy	1.99	1.92	0.738	0.838	1.19	2.91	2.71	2.58	2.6	3.09	2.76	3.06	3.05
Ho	0.32	0.31	0.14	0.16	0.21	0.58	0.53	0.51	0.52	0.63	0.55	0.62	0.62
Er	0.72	0.71	0.36	0.43	0.53	1.56	1.43	1.35	1.41	1.69	1.47	1.65	1.65
Tm	0.09	0.09	0.05	0.07	0.07	0.23	0.21	0.20	0.21	0.25	0.22	0.24	0.24
Yb	0.56	0.56	0.37	0.44	0.47	1.51	1.36	1.33	1.33	1.62	1.42	1.58	1.55
Lu	0.08	0.09	0.06	0.07	0.07	0.23	0.21	0.20	0.20	0.25	0.20	0.24	0.23
Hf	1.96	1.94	1.96	2.60	2.16	0.92	0.72	1.13	0.85	0.73	0.60	0.72	0.76
Ta	0.13	0.19	0.13	0.16	0.16	0.19	0.18	0.17	0.20	0.22	0.18	0.20	0.18
W	0.14	0.02	0.01	0.01	0.01	0.20	0.16	0.15	0.14	0.15	0.15	0.14	0.14
Tl	0.18	0.19	0.16	0.19	0.22	0.14	0.11	0.12	0.12	0.06	0.05	0.06	0.06
Pb	14.50	14.60	13.80	14.30	15.70	9.40	8.97	9.69	10.50	10.40	9.57	10.70	10.00
Bi	0.02	0.02	0.01	0.02	0.02	0.03	0.02	0.02	0.03	0.02	0.02	0.03	0.02
Th	6.40	6.80	1.53	1.28	2.67	1.54	1.27	1.22	1.42	1.19	1.15	1.13	1.11
U	1.42	1.03	0.363	0.461	0.589	0.538	0.356	0.349	0.419	0.358	0.334	0.322	0.31

Supplementary Table 1 continued

Sample No.	20WL-10	20WL-12	20WL-15	20WL-16	20WL-19	23ML-14	23ML-15	20ML-01	20ML-12	20ML-21	20ML-24
Rock type			tonalite			gabbro		hornblendite		pyroxene hornblendite	
Location			Wolong			Milin		Milin		Milin	
Crustal layer			MCC					LCC			
					Major elements (wt. %)						
SiO_2	57.01	57.26	56.44	54.34	55.31	43.19	43.84	44.62	44.75	41.60	42.01
TiO_2	0.79	0.73	0.65	0.75	0.76	1.48	1.38	1.06	0.50	2.10	2.03
Al2O3	17.85	17.88	18.07	18.39	18.21	19.23	18.96	8.83	22.01	14.16	14.04
$Fe_2O_3^T$	7.79	7.64	8.06	8.81	8.27	14.18	13.56	11.43	10.64	12.67	12.30
MnO	0.12	0.12	0.13	0.15	0.13	0.17	0.19	0.17	0.12	0.12	0.12
MgO	3.43	3.39	3.40	3.86	3.75	7.19	7.27	17.41	7.48	13.83	14.22
CaO	6.49	7.03	7.25	7.64	7.13	10.72	11.40	12.09	12.41	10.92	10.94
Na_2O	3.57	3.68	3.82	3.70	3.71	2.09	2.16	1.51	1.18	2.52	2.50
K_2O	1.59	1.29	0.94	1.31	1.38	0.35	0.31	0.30	0.13	0.57	0.55
P_2O_5	0.19	0.19	0.20	0.21	0.20	0.11	0.08	0.02	0.01	0.02	0.02
LOI	0.70	0.46	0.63	0.64	0.50	1.16	0.82	1.85	0.70	1.36	0.98
Total	99.69	99.81	99.75	99.94	99.49	99.87	99.96	99.29	100.07	99.87	99.71
Mg#	46.6	46.8	45.5	46.5	47.3	50.1	51.5	75.1	58.2	68.4	69.6
					Trace elements (μg/g)						
Li	22.2	16.4	16.5	16.5	9.88	6.56	4.39	5.4	3.47	9.15	8.27
Be	0.87	0.85	0.95	0.95	0.85	0.41	0.35	0.16	0.12	0.26	0.19
Sc	16.9	18.4	20.4	24.3	21.5	39	36.3	64.3	19.4	63.1	61
V	150	142	154	158	188	372	330	299	294	474	446
Cr	8.27	7.7	8.49	10.7	10.3	53.2	58.4	1096	57.2	36.6	43.9

Co	23.4	21.6	22.8	24.9	24.3	51.7	44.6	72.6	47.7	69.7	69.1
Ni	19.4	20.5	20.9	22.5	22.5	44.5	47.3	357	117	129	135
Cu	75.9	54.1	41	76.3	54.5	157	120	58.9	98	144	138
Zn	84.8	78	83.9	89.8	89	117	137	81.9	60.7	110	103
Ga	17.9	17.0	18.5	19.3	18.4	21.9	21.3	10.6	17.9	14.9	15.1
Ge	1.01	1.00	1.13	1.26	1.15	4.57	4.45	1.56	1.14	1.45	1.52
As	1.50	0.69	1.84	1.66	2.58	2.44	1.20	5.16	0.63	1.01	1.37
Rb	45	32.4	20.5	30.5	36.1	1.94	1.26	4.45	1.74	2.81	3.27
Sr	517	503	545	532	545	530	553	161	646	303	284
Y	11.3	13.8	14.5	22.9	16.1	25.2	25.2	15.1	4.45	19.1	20.9
Zr	66.6	44.3	71.8	64	50.1	11.9	12.8	16.9	4.74	22.3	22.5
Nb	3.18	2.94	2.68	3.95	3.3	1.66	1.57	0.883	0.127	1.43	1.42
Mo	0.384	0.336	0.206	0.290	0.789	0.040	0.026	0.253	0.134	0.084	0.114
Ag	0.140	0.165	0.126	0.188	0.148	0.052	0.054	0.052	0.066	0.093	0.100
Cd	0.072	0.083	0.080	0.121	0.086	0.086	0.095	0.077	0.051	0.101	0.087
In	0.033	0.041	0.042	0.057	0.044	0.093	0.095	0.058	0.027	0.081	0.086
Sn	0.687	0.695	0.682	0.956	0.761	0.454	0.425	0.644	0.185	0.904	0.976
Sb	0.066	0.040	0.052	0.074	0.041	0.112	0.032	0.119	0.017	0.112	0.077
Cs	2.65	1.31	1.36	1.53	1.89	0.08	0.10	0.08	0.37	0.07	0.06
Ba	361	292	242	270	315	117	86.3	43.5	34.2	72.6	66.5
La	12	11.1	11.4	14.1	11.9	4.31	4.13	1.43	0.813	1.84	1.84
Ce	23.2	23.1	24.4	31.4	24.8	13.7	12.3	4.82	1.81	7.07	6.87
Pr	2.76	2.82	2.97	4.12	3.05	2.49	2.21	0.932	0.27	1.35	1.43
Nd	11.2	12	12.2	17.9	13.1	14.1	13.1	5.49	1.63	8.65	8.76
Sm	2.24	2.59	2.68	4.23	2.91	4.26	4.17	2.04	0.498	2.98	3.21
Ti	4752	4351	3920	4498	4578	8875	8267	6347		12571	12150

Eu	0.847	0.809	0.862	1.1	0.906	1.39	1.38	0.552	0.213	0.831	0.825
Gd	1.95	2.38	2.45	3.75	2.63	4.3	4.36	1.98	0.498	2.87	2.88
Tb	0.316	0.385	0.388	0.649	0.433	0.707	0.735	0.359	0.095	0.51	0.547
Dy	1.95	2.38	2.34	4.02	2.64	4.49	4.57	2.45	0.659	3.43	3.74
Ho	0.40	0.49	0.48	0.79	0.54	0.93	0.94	0.53	0.14	0.69	0.74
Er	1.09	1.32	1.34	2.13	1.45	2.57	2.55	1.38	0.37	1.79	1.89
Tm	0.16	0.19	0.21	0.31	0.22	0.37	0.36	0.20	0.06	0.25	0.27
Yb	1.10	1.28	1.36	2.03	1.48	2.35	2.28	1.19	0.37	1.47	1.64
Lu	0.17	0.19	0.20	0.30	0.23	0.34	0.32	0.17	0.06	0.21	0.23
Hf	1.78	1.29	1.93	1.86	1.46	0.78	0.77	0.74	0.22	1.02	1.11
Ta	0.22	0.19	0.16	0.25	0.21	0.11	0.09	0.06	0.02	0.09	0.10
W	0.20	0.11	0.11	0.16	0.16	/	/	0.05	/	0.07	0.06
Tl	0.20	0.14	0.08	0.14	0.15	0.00	/	0.01	0.02	0.02	0.02
Pb	9.48	9.16	8.34	9.09	9.28	1.48	1.32	1.14	0.75	1.45	0.90
Bi	0.04	0.02	0.02	0.02	0.02	0.02	0.02	/	/	/	0.01
Th	1.85	1.44	1.57	1.63	1.87	0.04	0.04	0.15	0.04	0.07	0.09
U	0.533	0.411	0.486	0.51	0.536	0.013	0.014	0.061	0.035	0.044	0.049

Supplementary Table 2: $\delta^{98/95}$Mo and Mo concentrations of Gangdese arc crust section samples.

Sample	Rock type	Represent crustal layer	SiO_2 (wt.%)	Mo (μg/g)	$\delta^{98/95}$Mo (‰)	2 s.e.
20WL-01	granite	UCC	68.31	0.12	−0.17	0.07
20WL-02			68.79	0.05	−0.12	0.07
20WL-04			68.30	0.08	−0.09	0.07
20WL-07			68.56	0.07	−0.14	0.06
20WL-09			67.78	0.07	−0.16	0.07
20LL-01	medium-coarse grained quartz diorite	MCC	54.79	0.3	+0.16	0.07
20LL-03			56.3	0.53	+0.17	0.06
20LL-05			55.71	0.47	−0.20	0.07
20LL-07			56.95	0.43	+0.27	0.04
20LL-09	fine-grained quartz diorite		53.3	0.64	−0.06	0.05
20LL-10			53.14	0.62	−0.37	0.08
20LL-13			53.37	0.61	−0.32	0.07
20LL-16			53.07	0.66	+0.01	0.06
20WL-10	tonalite		57.01	0.29	−0.20	0.06
20WL-12			57.26	0.23	−0.15	0.06
20WL-15			56.44	0.12	−0.04	0.07
20WL-16			54.34	0.24	−0.07	0.08
20WL-19			55.31	0.55	−0.04	0.05
23ML-14	gabbro	LCC	43.19	0.03	−0.38	0.04
23ML-15			43.84	0.04	−0.41	0.04
20ML-01	hornblendite		44.62	0.06	+0.20	0.05
20ML-12			44.75	0.04	−0.07	0.05
20ML-21	pyroxene hornblendite		41.60	0.04	−0.19	0.08
20ML-24			42.01	0.05	−0.17	0.04

Standards	Mo (μg/g)	2 s.d.	$\delta^{98/95}$Mo (‰)	2 s.d.	n	References
AGV-2	1.99	0.03	−0.15	0.03	4	This study
AGV-2	1.96	0.05	−0.15	0.01	3	Willbold et al.[35]
W-2a	0.44	0.05	−0.04	0.06	5	This study
W-2a	0.41	0.03	−0.03	0.04	13	Chen et al.[36]
seawater	0.01	0.001	+2.06	0.03	4	This study
seawater	0.01	0.001	+2.02	0.06	20	Zhao et al.[37]
Blank	0.00039	0.00037			7	This study

Note: Each solution was measured once, with more than 60 cycles per measurement. Seawater in this study is IAPSO Atlantic seawater.

Supplementary Table 3: Mo concentrations of different reservoirs used to calculate and evaluate Mo isotope composition of bulk continental crust.

Rock type	Represent the reservoir	Mo concentrations (μg/g)			2 s.d.	95% s.e.	Number	References
		Arithmetic average	Median	Highest relative probability value				
**Intrusion**								
SiO_2 ≥63 wt.%	UCC	0.915	0.700	0.519	1.720	0.137	15	this study; Xue et al.[38]; Shen et al.[39]; Kaufmann et al.[40]; Fan et al.[41]; Yang et al.[42]; Greber et al.[43]; Fan et al.[44]; Xue et al.[45]; Xia et al.[46]; Fan et al.[47]
52 wt.% < SiO_2 < 63 wt.%	MCC	1.066	0.649	0.549	1.822	0.254	52	this study; Xue et al.[38]; Shen et al.[39]; Fan et al.[41]; Yang et al.[42]; Fan et al.[47]; Yang et al.[48]
SiO_2 ≤ 52 wt.%	LCC	0.328	0.174	0.124	0.688	0.062	119	this study; Sun et al.[27]; Fan et al.[44]; Yang et al.[48]; Zhang et al.[49]; Fang et al.[50]; Storck et al.[51]; Nebel-Jacobsen et al.[52]; Liang et al.[53]; Fang et al.[54]
**Mid-ocean ridge basalt (MORB)**	oceanic crust	0.574	0.420	0.357	1.011	0.101	99	McCoy-West et al.[32]; Liang et al.[53]; Cai et al.[55]; Chen et al.[56]; Hin et al.[57]; Bezard et al.[58]
**Diamictites, loess and clay**	UCC	0.880	0.720		1.260	0.189	45	Wang et al.[16]; Greaney et al.[24]

Supplementary Table 4: Mo isotope compositions of different reservoirs used to calculate and evaluate Mo isotope composition of the bulk continental crust

Rock type	Represent the reservoir	$\delta^{98/95}$Mo (‰)			2 s.d.	95% s.e.	Number	References
		Arithmetic average	Median	Highest relative probability value				
**Intrusion**								
SiO_2 ≥63 wt.%	UCC	−0.079	−0.070	−0.014	0.762	0.060	159	this study; Xue et al.[38]; Shen et al.[39]; Kaufmann et al.[40]; Fan et al.[41]; Yang et al.[42]; Greber et al.[43]; Fan et al.[44]; Xue et al.[45]; Xia et al.[46]; Fan et al.[47]
52 wt.% < SiO_2 < 63 wt.%	MCC	−0.123	−0.130	−0.139	0.579	0.081	52	this study; Xue et al.[38]; Shen et al.[39]; Fan et al.[41]; Yang et al.[42]; Fan et al.[47]; Yang et al.[48]
SiO_2 ≤ 52 wt.%	LCC	−0.165	−0.172	−0.179	0.414	0.038	119	this study; Sun et al.[27]; Fan et al.[44]; Yang et al.[48]; Zhang et al.[49]; Fang et al.[50]; Storck et al.[51]; Nebel-Jacobsen et al.[52]; Liang et al.[53]; Fang et al.[54]
**Mid-ocean ridge basalt (MORB)**	oceanic crust	−0.175	−0.180	−0.181	0.278	0.028	99	McCoy-West et al.[32]; Liang et al.[53]; Cai et al.[55]; Chen et al.[56]; Hin et al.[57]; Bezard et al.[58]
**Diamictites, loess and clay**	UCC	−0.226	−0.070	−0.003	0.79	0.119	45	Wang et al.[16]; Greaney et al.[24]

Supplementary Table 5: Estimated densities and Mo concentrations of the upper, middle, and lower continental crust.

Reservoirs	Density(kg/m^3)	Mo concentration (μg/g)		
upper continental crust	2700	1.1	1.4	1.5
middle continental crust	2905	0.6	0.6	0.8
lower continental crust	3006	0.6	0.6	0.8
bulk continental crust		0.8	1.1	1
References	Hacker et al.[34]	Rudnick and Gao[20]	Wedepohl[28]	Taylor and Mcleanan[29]

Supplementary Table 6: Mo isotope compositions of the bulk continental crust using median of $\delta^{98/95}$Mo under different continental crust models and Mo concentrations.

Mo concentration sources	Continental crust models						Average $\delta^{98/95}$Mo (‰)	2 s.d
	Rudnick and Gao[20]		Huang et al.[33]		Hacker et al.[34]			
	$\delta^{98/95}$Mo (‰)	95%s.e.	$\delta^{98/95}$Mo (‰)	95%s.e.	$\delta^{98/95}$Mo (‰)	95%s.e.		
**Rudnick and Gao[20]**	−0.120	0.053	−0.109	0.055	−0.120	0.048	**−0.116 (a)**	**0.011**
**Taylor and McLennan[29]**	−0.120	0.053	−0.109	0.055	−0.119	0.049		
**Compilation of intrusions**	−0.111	0.057	−0.105	0.058	−0.113	0.050	−0.110 (b)	0.009
**Average**		2 s.d.		2 s.d.		2 s.d.		
**$\delta^{98/95}$Mo (‰) (c)**	−0.117	0.010	−0.108	0.005	−0.118	0.007		

Note: (a) The average $\delta^{98/95}$Mo of the bulk continental crust (n = 6) calculated using estimated Mo concentrations[20,29] under three crustal depth models[20,33,34]. (b) The average $\delta^{98/95}$Mo of the bulk continental crust (n = 3) calculated using compiled Mo concentrations of intrusive rocks under three crustal depth models[20,33,34]. (c) The average $\delta^{98/95}$Mo of the bulk continental crust calculated using estimated[20,29] and complied Mo concentrations under the same crustal depth model. The complied Mo concentration is the median of Mo concentration.

Supplementary Table 7: Mo concentration and isotopes compositions of geochemical reservoirs used in mass balance calculations

Reservoir	Density (kg/m^3)	Mass(kg)	Volume (m^3)	Mo (μg/g)	$\delta^{98/95}Mo$ (‰)
chondrites				ca.1.7 (a)	−0.154 ± 0.013 (b, c)
Bulk silicate Earth		$4.0603c\times10^{24}$ (d)		0.047 ± 0.019 (e)	−0.154 (d)
Mantle		4.0343×10^{24} (d)			
Depleted mantle		Varied		0.025 ± 0.007 (f)	−0.204 ± 0.008 (d)
	2886 (g)	2.43×10^{22} (g)	8.42×10^{18}		
Modern continental crust	2856 (h)	2.04×10^{22} (h)	7.14×10^{18}		
	2866 (i)	2.41×10^{22} (i)	8.41×10^{18}		
Modern oceanic crust	2890 (j)	6.83×10^{21}	2.36×10^{18} (k)		

References for other parameters as follows: (a) McDonough[59]; (b) Burkhardt et al.[60]; (c) Liang et al.[53]; (d) McCoy-West et al.[32]; (e) Palme and O'Neill[61]; (f) Salters and Stracke[62]; (g) Rudnick and Gao[20]; (h) Huang et al.[33]; (i) Hacker et al.[34]; (j) Carlson and Raskin[63]; (k) Cogley[64].

Supplementary References

1. Ji W-Q, Wu F-Y, Chung S-L, Li J-X, Liu C-Z. Zircon U–Pb geochronology and Hf isotopic constraints on petrogenesis of the Gangdese batholith, southern Tibet. Chemical Geology 262, 229-245 (2009).
2. Ma L, et al. Early Late Cretaceous (ca. 93Ma) norites and hornblendites in the Milin area, eastern Gangdese: Lithosphere–asthenosphere interaction during slab roll-back and an insight into early Late Cretaceous (ca. 100–80Ma) magmatic "flare-up" in southern Lhasa (Tibet). Lithos 172-173, 17-30 (2013).
3. Zhu D-C, et al. The Lhasa Terrane: Record of a microcontinent and its histories of drift and growth. Earth and Planetary Science Letters 301, 241-255 (2011).
4. Ma L, et al. Late Cretaceous (100–89Ma) magnesian charnockites with adakitic affinities in the Milin area, eastern Gangdese: Partial melting of subducted oceanic crust and implications for crustal growth in southern Tibet. Lithos 175-176, 315-332 (2013).
5. Kapp P, Yin A, Harrison TM, Ding L. Cretaceous-Tertiary shortening, basin development, and volcanism in central Tibet. GSA Bulletin 117, 865-878 (2005).
6. Guo L, Jagoutz O, Shinevar WJ, Zhang H-F. Formation and composition of the Late Cretaceous Gangdese arc lower crust in southern Tibet. Contributions to Mineralogy and Petrology 175, 58 (2020).
7. Quanru G, et al. The Eastern Himalayan syntaxis: major tectonic domains, ophiolitic mélanges and geologic evolution. Journal of Asian Earth Sciences 27, 265-285 (2006).
8. Xu Z, Ji S, Cai Z, Zeng L, Geng Q, Cao H. Kinematics and dynamics of the Namche Barwa Syntaxis, eastern Himalaya: Constraints from deformation, fabrics and geochronology. Gondwana Research 21, 19-36 (2012).
9. Zhang Z, Ding H, Palin RM, Dong X, Tian Z, Chen Y. The lower crust of the Gangdese magmatic arc, southern Tibet, implication for the growth of continental crust. Gondwana Research 77, 136-146 (2020).
10. Guo L, Zhang H-F, Harris N, Luo B-J, Zhang W, Xu W-C. Tectonic erosion and crustal relamination during the India-Asian continental collision: Insights from Eocene magmatism in the southeastern Gangdese belt. Lithos 346-347, 105161 (2019).
11. Guo L, Zhang H-F, Harris N, Pan F-B, Xu W-C. Late Cretaceous (~81Ma) high-temperature metamorphism in the southeastern Lhasa terrane: Implication for the Neo-Tethys ocean ridge subduction. Tectonophysics 608, 112-126 (2013).
12. Zhang Z, Dong X, Xiang H, He Z, Liou JG. Metagabbros of the Gangdese arc root, south Tibet: Implications for the growth of continental crust. Geochimica et Cosmochimica Acta 143, 268-284 (2014).
13. Zhang Z, Zhao G, Santosh M, Wang J, Dong X, Shen K. Late Cretaceous charnockite with adakitic affinities from the Gangdese batholith, southeastern Tibet: Evidence for Neo-Tethyan mid-ocean ridge subduction? Gondwana Research 17, 615-631 (2010).
14. Wen D-R, et al. Late Cretaceous Gangdese intrusions of adakitic geochemical characteristics, SE Tibet: Petrogenesis and tectonic implications. Lithos 105, 1-11 (2008).
15. Zheng Y-F, Zhao Z-F, Chen L, Tang Y-W. Geochemical evidence for the production of granitoids through reworking of the juvenile mafic arc crust in the Gangdese orogen, southern Tibet. GSA Bulletin 132, 1347-1364 (2019).
16. Wang Z, et al. Fe (hydro) oxide controls Mo isotope fractionation during the weathering of granite. Geochimica et Cosmochimica Acta 226, 1-17 (2018).
17. Ma J-L, Wei G-J, Xu Y-G, Long W-G, Sun W-D. Mobilization and re-distribution of major and trace elements during extreme weathering of basalt in Hainan Island, South China. Geochimica et

Cosmochimica Acta 71, 3223-3237 (2007).
18. Russo SC, González-Álvarez I, Cocker HA, McCoy-West AJ. The fundamentals of rare earth element ion adsorption clay deposits: A mineral systems approach for exploration. Journal of Geochemical Exploration 278, 107845 (2025).
19. Ohta T, Arai H. Statistical empirical index of chemical weathering in igneous rocks: A new tool for evaluating the degree of weathering. Chemical Geology 240, 280-297 (2007).
20. Rudnick RL, Gao S. Composition of the Continental Crust. In: Treatise on Geochemistry) (2014).
21. Clift PD, Vannucchi P, Morgan JP. Crustal redistribution, crust–mantle recycling and Phanerozoic evolution of the continental crust. Earth-Science Reviews 97, 80-104 (2009).
22. Hawkesworth C, Cawood P, Dhuime B. Continental growth and the crustal record. Tectonophysics 609, 651-660 (2013).
23. Rudnick R, Gao S. Composition of the continental crust. Treatise on Geochemistry, vol. 3. Treat Geochem, 1-64 (2003).
24. Greaney AT, Rudnick RL, Romaniello SJ, Johnson AC, Gaschnig RM, Anbar AD. Molybdenum isotope fractionation in glacial diamictites tracks the onset of oxidative weathering of the continental crust. Earth and Planetary Science Letters 534, 116083 (2020).
25. Tian Y, et al. Petrogenesis and geodynamic mechanisms of the Late Cretaceous magmatic 'flare-up'in the southern Lhasa Terrane, Tibet. Lithos 424, 106766 (2022).
26. Sun S-S, McDonough WF. Chemical and isotopic systematics of oceanic basalts: implications for mantle composition and processes. Geological Society, London, Special Publications 42, 313-345 (1989).
27. Sun J-L, Bai Z-J, Zhu W-G, Liu X. Molybdenum isotopes record dehydrated slab components input to arc magmatism in subduction zones. Geochimica et Cosmochimica Acta, 152-162 (2025).
28. Wedepohl KH. The composition of the continental crust. Geochimica et cosmochimica Acta 59, 1217-1232 (1995).
29. Taylor SR, McLennan SM. The continental crust: its composition and evolution. (1985).
30. Rudnick RL, Fountain DM. Nature and composition of the continental crust: a lower crustal perspective. Reviews of geophysics 33, 267-309 (1995).
31. Gao S, et al. Chemical composition of the continental crust as revealed by studies in East China. Geochimica et cosmochimica acta 62, 1959-1975 (1998).
32. McCoy-West AJ, et al. Extensive crustal extraction in Earth's early history inferred from molybdenum isotopes. Nature Geoscience 12, 946-951 (2019).
33. Huang Y, Chubakov V, Mantovani F, Rudnick RL, McDonough WF. A reference Earth model for the heat-producing elements and associated geoneutrino flux. Geochemistry, Geophysics, Geosystems 14, 2003-2029 (2013).
34. Hacker BR, Kelemen PB, Behn MD. Continental Lower Crust. Annual Review of Earth and Planetary Sciences 43, 167-205 (2015).
35. Willbold M, et al. High-precision mass-dependent molybdenum isotope variations in magmatic rocks determined by double-spike MC-ICP-MS. Geostandards and Geoanalytical Research 40, 389-403 (2016).
36. Chen S, et al. Molybdenum systematics of subducted crust record reactive fluid flow from underlying slab serpentine dehydration. Nat Commun 10, 4773 (2019).
37. Zhao PP, et al. Molybdenum Mass Fractions and Isotopic Compositions of International Geological Reference Materials. Geostandards and Geoanalytical Research 40, 217-226 (2016).
38. Xue Q, Zhang L, Chen S, Li C, Li T, Sun W. The source and ore-forming processes of post-collisional Qulong porphyry Cu-Mo deposit in Tibet constrained by Mo isotopes. Chemical Geology 652, 122025

(2024).
39. Shen J, et al. Molybdenum isotope tracing petrogenesis of adakitic rocks and associated ore-forming process. Geochimica et Cosmochimica Acta 300, 296-317 (2021).
40. Kaufmann AKC, Pettke T, Wille M. Molybdenum isotope fractionation at upper-crustal magmatic-hydrothermal conditions. Chemical Geology 578, 120319 (2021).
41. Fan JJ, et al. Molybdenum and Boron Isotopic Compositions of Porphyry Cu Mineralization-Related Adakitic Rocks in Central-Eastern China: New Insights Into their Petrogenesis and Crust-Mantle Interaction. Journal of Geophysical Research: Solid Earth 125, e2020JB020474 (2020).
42. Yang J, Barling J, Siebert C, Fietzke J, Stephens E, Halliday AN. The molybdenum isotopic compositions of I-, S- and A-type granitic suites. Geochimica et Cosmochimica Acta 205, 168-186 (2017).
43. Greber ND, Pettke T, Nägler TF. Magmatic–hydrothermal molybdenum isotope fractionation and its relevance to the igneous crustal signature. Lithos 190-191, 104-110 (2014).
44. Fan J-J, et al. Extreme Mo isotope variations recorded in high-SiO2 granites: Insights into magmatic differentiation and melt–fluid interaction. Geochimica et Cosmochimica Acta 334, 241-258 (2022).
45. Xue Q, et al. Tracing black shales in the source of a porphyry Mo deposit using molybdenum isotopes. Geology 51, 688-692 (2023).
46. Xia Y-S, et al. Fluid-fluxed melting of juvenile lower crust traced by molybdenum isotopes. Geology 53, 125-129 (2024).
47. Fan JJ, et al. Mo isotope evidence for the significance of subducted continental crust in formation of post-collisional porphyry Cu deposits. Chemical Geology 680, 122683 (2025).
48. Yang Q-C, Fang W, Dai L-Q, Zhao Z-F, Sun G-C, Gong B. Mo-Mg isotopes trace the role of serpentinite in generating arc magmatism. Chemical Geology 654, 122074 (2024).
49. Zhang L, Li J, Zhang Y, Peng B-Y, Wang Z-B, Ren Z-Y. Molybdenum isotopic fractionation in the Panzhihua mafic layered intrusion in the Emeishan large igneous province, southwest China. American Mineralogist 109, 628-632 (2024).
50. Fang W, Dai L-Q, Fu X, Zhao Z-F, Gong B, Zheng Y-F. Molybdenum isotopes record recycling of subducting sediment in active continental margin, Northeast China. Chemical Geology 627, 121460 (2023).
51. Storck J-C, Greber ND, Duarte JFV, Lanari P, Tiepolo M, Pettke T. Molybdenum and titanium isotopic signatures of arc-derived cumulates. Chemical Geology 617, 121260 (2023).
52. Nebel-Jacobsen Y, Wille M, Ivanic T, Nebel O. Molybdenum isotope systematics in cumulate rock of the 2.8 Windimurra layered intrusion: A test for igneous differentiation and the composition of the Archean mantle. Precambrian Research 355, 106087 (2021).
53. Liang Y-H, et al. Molybdenum isotope fractionation in the mantle. Geochimica et Cosmochimica Acta 199, 91-111 (2017).
54. Fang W, Dai L-Q, Zheng Y-F, Zhao Z-F. Molybdenum isotopes in mafic igneous rocks record slabmantle interactions from subarc to postarc depths. Geology 51, 3-7 (2022).
55. Cai Y-X, et al. Contribution of recycled oceanic crust to the extremely light molybdenum isotopic compositions of mid-ocean ridge basalts from the South China Sea. Chemical Geology 646, 121916 (2024).
56. Chen S, Sun P, Niu Y, Guo P, Elliott T, Hin RC. Molybdenum isotope systematics of lavas from the East Pacific Rise: Constraints on the source of enriched mid-ocean ridge basalt. Earth and Planetary Science Letters 578, 117283 (2022).
57. Hin RC, et al. The influence of crustal recycling on the molybdenum isotope composition of the

Earth's mantle. Earth and Planetary Science Letters 595, 117760 (2022).
58. Bezard R, Fischer-Gödde M, Hamelin C, Brennecka GA, Kleine T. The effects of magmatic processes and crustal recycling on the molybdenum stable isotopic composition of Mid-Ocean Ridge Basalts. Earth and Planetary Science Letters 453, 171-181 (2016).
59. McDonough W. Compositional model for the Earth's core. Treatise on geochemistry 2, 568 (2003).
60. Burkhardt C, Hin RC, Kleine T, Bourdon B. Evidence for Mo isotope fractionation in the solar nebula and during planetary differentiation. Earth and Planetary Science Letters 391, 201-211 (2014).
61. Palme H, O'Neill HSC. Cosmochemical Estimates of Mantle Composition. In: Treatise on Geochemistry) (2014).
62. Salters VJM, Stracke A. Composition of the depleted mantle. Geochemistry, Geophysics, Geosystems 5, Q05B07 (2004).
63. Carlson R, Raskin G. Density of the ocean crust. Nature 311, 555-558 (1984).
64. Cogley JG. Continental margins and the extent and number of the continents. Reviews of Geophysics 22, 101-122 (1984).
